# Seroprevalence and Potential Risk Factors for *Helicobacter pylori* Infection in Brazilian Children

**DOI:** 10.1111/j.1523-5378.2010.00766.x

**Published:** 2010-08

**Authors:** Vitor Camilo Cavalcante Dattoli, Rafael Valente Veiga, Sergio Souza da Cunha, Lain Carlos Pontes-de-Carvalho, Maurício Lima Barreto, Neuza Maria Alcântara-Neves

**Affiliations:** * Health Science Institute, Federal University of BahiaBahia, Brazil; † Health Science Center, Federal University of PernambucoPernambuco, Brazil; ‡ Research Center Gonçalo Moniz, Oswaldo Cruz FoundationBahia, Brazil; § Community Health Institute, Federal University of BahiaBahia, Brazil

**Keywords:** *Helicobacter pylori*, seroepidemiology, risk factors, children, Brazil

## Abstract

**Background:**

*Helicobacter pylori* infection has been proved to be of great relevance to public health in unindustrialized countries, especially in low socioeconomic groups. Poor hygiene, deficient sanitation, and crowded conditions have been reported as risk factors for this infection. In this work, we investigated whether social and demographic characteristics were associated with anti-*H. pylori* IgG antibodies in 1104 children aged 4–11 years old from Salvador, a large city located in northeastern Brazil.

**Methods:**

Standardized questionnaires were used to obtain social, demographic, and environmental data for the studied population in two periods of time (from 1997 to 2003 and in 2005). Anti-*H. pylori* IgG antibodies were assessed by indirect enzyme-linked immunosorbent assay in 2005.

**Results:**

Anti-*H. pylori* IgG antibody was present in 28.7% of the children. Among the studied variables, the following were positively associated with the presence of anti-*H. pylori* antibodies in multivariable analyses: age above 8 years old (OR = 1.72, 95% CI = 1.23–2.40), a larger sibling number (OR = 1.66, 95% CI = 1.26–2.18), nursery attendance (OR = 1.49, 95% CI = 1.04–2.12), location of the house at an unpaved street (OR = 2.03, 95% CI = 1.44–2.87) and absence of a flush toilet (OR = 1.32, 95% CI = 1.00–1.74).

**Conclusion:**

Our data show that *H. pylori* infection in children from a major Brazilian city is associated with variables indicative of a crowded environment and deficient sanitation/habitation conditions, leading to the conclusion that improvements in hygiene and social conditions may protect children against this infection.

*Helicobacter pylori* is a spiral Gram negative bacterium that colonizes the human stomach [1] and is the main cause of peptic ulcer [2], gastric adenocarcinoma and primary gastric lymphoma [1,3] in adulthood. It has been found to infect more than half of the world’s population [4]. The presence of *H. pylori* in saliva, dental plaque [5], and feces [6] and the lack of significant evidence of nonhuman or environmental reservoirs [7] indicate that person-to-person spreading is probably a major transmission mechanism of this infection. There is also clear evidence that *H. pylori* infection is primarily acquired early in life [8,9]. Poor hygiene standards, crowded households and deficient sanitation are important to both acquisition of infection in childhood and spreading of the disease within households [10,11].

The improvement of hygiene conditions has significantly decreased the prevalence of this infection in many parts of North America and Europe [12]. Unfortunately, very high disease prevalence persist in developing countries [13], where *H. pylori* seroprevalences may exceed 50% in children and over 90% in adults [14–18].

In Brazil, epidemiological studies of *H. pylori* infection have revealed high prevalences of the infection among adults [19,20], similar to the results of studies in other developing countries [16]. Moreover, Braga et al. have reported a 40% seroprevalence in children under 6 years of age from a low income population [21].

Considering that the epidemiology of this infection is still quite poorly studied in Brazil, the main objective of this study was to estimate the seroprevalence and potential risk factors for *H. pylori* infection in a large children cohort from Salvador, a city located in northeastern Brazil. A seroprevalence of 28.7% was found. In addition, conditions indicative of poor sanitation/habitation and of crowded households were significantly associated with a positive serology for anti-*H. pylori* antibodies.

## Materials and Methods

### Study Population

This prospective study was conducted in the city of Salvador, in the Brazilian Northeast region, which has a population of 2.8 million people. Three baseline surveys were carried out in 1997, 2000, and 2003, allowing different children, born between 1994 and 2001, to be recruited and then followed-up. These three surveys were part of a study aimed at evaluating the impact of a sanitation programme on the incidence of childhood diarrhea [22]. In these baseline surveys, demographic and social data, which are used in this study, were collected using a standardized questionnaire. In 2005, 1445 of these children were resurveyed, as detailed elsewhere [23]. Briefly, social and demographic information were recollected and the presence of specific antibodies against several pathogens, including *H. pylori*, in sera prepared from small volume blood samples, was investigated by enzyme-linked immunosorbent assay (ELISA). The data obtained from these children were used to evaluate whether the presence of positive serology in 2005 was associated with exposures to potential risk factors assessed in the 2005 and/or in the previous (1997, 2000, and 2003) surveys. Informed consent was obtained from the children’s parents or guardians. Ethical approval was granted by the Instituto de Saúde Coletiva at Universidade Federal da Bahia and the National Commission on Ethics in Research (CONEP), Brazil.

### Potential Risk Factors for *H. pylori* Infection

The following variables collected in the baseline surveys between 1997 and 2003 were analyzed as potential risk factors for *H. pylori* infection (an outcome that was revealed in the 2005 survey): treated piped water at home; flooded house during the rainy season; presence of a flush toilet; house served by a paved road; open sewage nearby; frequency of rubbish collection. The following variables from the 2005 survey were also investigated as potential risk factors: maternal schooling; meat intake (how often the child has eaten chicken, beef or pork); vegetable intake (how often the child has eaten vegetables); presence of older sibling, number of siblings; whether the child attended nursery; presence of rodents, flies, dogs or cats at home; treated piped water at home; house served by a paved road; type of waste disposal system. The child’s sex and age (in 2005) were treated as *a priori* confounders.

### Serological Detection of Anti-*H. pylori* IgG

The presence of these antibodies in blood samples collected in 2005 was determined by ELISA using a commercially available kit (Diamedix, Miami, FL, USA), following the directions provided by the supplier. The cut-off was determined by an index value obtained by the ratio of sample absorbance to the absorbance of a calibrator (a solution containing human serum or defibrinated plasma, with IgG antibodies weakly reactive with *H. pylori* and 0.1% sodium azide). A ratio >1.1 was considered positive. Borderline subjects were removed from the analysis.

### Statistical Analysis

Only children for whom complete data were available were included in the analysis. We first performed a bivariable analysis between each potential risk factor and outcome. Second, we built a multivariable model with standard logistic regression including only significant variables from the bivariable analysis. Then, we assessed each nonsignificant variable a second time by including each one in the model (one at a time). If the variable remained nonsignificant, it was completely removed from the analysis. Each time that one variable became significant, it was kept in the model, and all remaining nonsignificant variables were reassessed each one at a time. This process was repeated until no variables remained to be assessed. The association between outcome and risk factors was estimated with odds ratio and 95% confidence interval.

## Results

Among the original 1445 children in the 2005 survey, 1104 had complete data sets and were used in the analysis. No differences were found between the excluded and the studied population in relation to the studied variables and outcome (data not shown). The prevalence of anti-*H. pylori* IgG antibodies was 28.7%. The characteristics of the studied population are shown in Table 1. The mean age plus the standard deviation was 6.8 ± 0.5 years old. Approximately, half (580) of the studied children were male (52.5%) and 347 (31.4%) of the mothers had completed a high school degree. With regard to opportunity of frequent contact with other children, 424 (38.4%) had two or more siblings, 683 (61.9%) had at least one older sibling and 174 (15.8%) had attended nursery school for some time. About 494 (44.7%) consumed meat frequently (≥3 times a week) and 866 (78.4%) consumed vegetables at least once a week. Most of the subjects reported the presence of rodents and flies at home. With respect to living conditions, 443 (40.1%) did not live in houses on streets with pavement, in any of the years of the surveys (from 1997 to 2005); 888 (80.4%) had treated piped water available at home in all investigated periods; 456 (37.5%) reported the absence of a toilet and 179 (16.2%) had no sewage system at home; 517 (46.8%) reported open sewage near the house; and 339 (30.7%) had their houses flooded during the rainy season.

**Table 1:** Sociodemographic and environmental characteristics of the study population: 1104 children analyzed

Study variables	n	(%)
Gender (male)	580	52.5
Age, years (mean, SD)	6.80	0.5
Mother’s schooling		
Illiterate or primary complete	516	46.9
Secondary education complete	241	21.8
High school complete	347	31.4
Number of siblings (≥2)	424	38.4
Older sibling (yes)	683	61.9
Nursery attendance (yes)	174	15.8
Smoking at home (yes)	308	27.9
Mother smoked during the child’s first year (yes)	120	10.9
Beef intake (≥3 times weekly)	494	44.7
Vegetable intake (≥1 times weekly)	866	78.4
Dog at home (yes)	438	39.7
Cat at home (yes)	190	17.2
Rodents in the home (yes)	629	57.0
Flies in the home (yes)	551	49.9
House served by a paved road		
Never	443	40.1
Only in 1997–2003	283	25.6
Only in 2005	75	6.8
1997–2003 and 2005	303	27.4
Treated piped water		
Never	33	3.0
Only in 1997–2003	49	4.4
Only in 2005	134	12.1
1997–2003 and 2005	888	80.4
Lack of a flush toilet	411	37.2
Lack of a sewage system	179	16.2
Presence of open sewer nearby (yes)	517	46.8
Existence of rubbish collection (<1 time per week)	842	76.3
Possibility of a flooded house^a^ (yes)	339	30.7

SD, standard deviation.

a If the house floods in the rainy season.

In the bivariable (uncontrolled) analysis, the following variables were significantly associated with an increased prevalence of *H. pylori* infection: age (OR = 1.53, 95% CI = 1.11–2.11); number of siblings (OR = 1.71, 95% CI = 1.31–2.23); nursery attendance (OR = 1.42, 95% CI = 1.01–2.00); meat intake (OR = 1.55, 95% CI = 1.05–2.31); whether the house was served by an unpaved road (OR = 1.71, 95% CI = 1.23–2.38); and the presence/absence of flush toilet at home (OR = 1.32, 95% CI = 1.01–1.73) (Tables 2 and 3).

**Table 2:** Association between sociodemographic characteristics and *Helicobacter pylori* infection, using bivariate analyses (1104 children)

	*H. pylori* infection 317 (28.7%)		
Study variables	n/N	%	OR (95% CI)^a^
Gender			
Female	143/524	27.3	1
Male	174/580	30.0	1.14 (0.88–1.48)
Age class			
<6	106/420	25.2	1
6–7	105/373	28.2	1.16 (0.85–1.59)*
≥8	106/311	34.1	1.53 (1.11–2.11)*
Mother’s schooling			
Illiterate or primary complete	159/516	30.8	1
Secondary education complete	67/341	27.8	0.87 (0.62–1.21)
High school complete	91/247	26.2	0.80 (0.59–1.08)
Number of siblings			
<2	166/680	24.4	1
≥2	151/424	35.6	1.71 (1.31–2.23)*
Older sibling			
No	115/421	27.3	1
Yes	202/683	29.6	1.12 (0.85–1.46)
Nursery attendance			
No	256/930	27.5	1
Yes	61/174	35.1	1.42 (1.01–2.00)*
Smoking at home (anyone)			
No	235/796	29.5	1
Yes	82/308	26.6	0.87 (0.65–1.16)
Mother smoked during the child’s first year			
No	275/984	27.9	1
Yes	42/120	35.0	1.39 (0.93–2.07)
Beef intake^b^			
Rare	41/187	21.9	1
1–2 times weekly	126/423	29.8	1.51 (1.01–2.26)*
≥3 times weekly	150/494	30.4	1.55 (1.05–2.31)*
Vegetable intake^c^			
≥1 times weekly	257/866	29.7	1
Rare	60/238	25.2	0.80 (0.58–1.11)

OR, odds ratio; CI, confidence interval.

* Significant value (*p* < .05).

a OR and CI 95% of crude analysis.

b How often did the child eat meat (chicken, beef, pork)?

c How often did the child eat vegetables?

**Table 3:** Associations between environmental characteristics and *Helicobacter pylori* infection, using bivariate analyses (1104 children)

	*H. pylori* infection 317 (28.7%)		
Study variables	n/N	%	OR (95% CI)^a^
Flies in the home			
No or rare	160/553	28.9	1
Yes	157/551	28.5	0.98 (0.75–1.27)
Rodents in the home			
No	137/475	28.8	1
Yes	180/629	28.6	1.00 (0.76–1.29)
Cat at home			
No	260/914	28.4	1
Yes	57/190	30.0	1.08 (0.77–1.52)
Dog at home			
No	196/666	29.4	1
Yes	121/438	27.6	0.92 (0.70–1.20)
Paved road (1997–2003 and 2005)			
All periods	72/303	23.8	1
One period	91/358	25.4	1.09 (0.77–1.56)
Neither period	154/443	34.8	1.71 (1.23–2.38)*
Piped water (1997–2003 and 2005)			
All periods	256/888	28.8	1
One period	51/183	27.9	0.95 (0.67–1.36)
Neither period	10/33	30.3	1.07 (0.50–2.29)
Flush toilet			
Yes	184/693	26.6	1
No	133/411	32.4	1.32 (1.01–1.73)*
Sewage system			
Yes	263/925	28.4	1
No	54/179	30.2	1.09 (0.77–1.54)
Open sewer nearby			
No	169/587	28.8	1
Yes	148/517	28.6	0.99 (0.76–1.29)
Rubbish collection			
≥1 per week	252/842	29.9	1
<1 per week	65/262	24.8	0.77 (0.56–1.06)
Flooded house			
No	222/765	29.0	1
Yes	95/339	28.0	0.95 (0.72–1.27)

OR, odds ratio; CI, confidence interval.

* Significant value (*p* < .05).

a OR and CI 95% of crude analysis.

The variables that kept a statistically significant association with *H. pylori* infection in the multivariable (final) model are shown in Table 4. A significant higher prevalence was found in older than in younger children (adjusted OR = 1.72, 95% CI = 1.23–2.40). The existence of two or more siblings (adjusted OR = 1.66, 95% CI = 1.26–2.18), or nursery attendance (adjusted OR = 1.49, 95% CI = 1.04–2.12) were also positively associated with *H. pylori* infection. Those children who lived in a house not served by a paved street in all studied years (1997–2005) had higher chance of being infected than children living in houses served by paved road at the whole period (adjusted OR = 2.03, 95% CI = 1.44–2.87). Finally, children living in a house without a flush toilet were proportionally more infected that those who lived in a house with flush toilet (adjusted OR = 1.32, 95% CI = 1.00–1.74).

**Table 4:** Multivariable analysis between exposure variables and *Helicobacter pylori* infection (1104 children)

	*H. pylori* infection 317 (28.7)		
Study variables	n/N	(%)	Final model OR (95% CI)
Gender			
Female	143/524	27.3	1
Male	174/580	30.0	1.11 (0.85–1.45)
Age			
≤5	106/420	25.2	1
6–7	105/373	28.2	1.08 (0.78–1.49)
≥8	106/311	34.1	1.72 (1.23–2.40)*
Number of siblings			
<2	166/680	24.4	1
≥2	151/424	35.6	1.66 (1.26–2.18)*
Nursery attendance			
No	256/930	27.5	1
Yes	61/174	35.1	1.49 (1.04–2.12)*
Paved road (1997–2003 and 2005)			
All periods	72/303	23.8	1
One period	91/358	25.4	1.14 (0.79–1.64)
Neither period	154/443	34.8	2.03 (1.44–2.87)*
Flush toilet			
Yes	184/693	26.6	1
No	133/411	32.4	1.32 (1.00–1.74)*

OR, odds ratio; CI, confidence interval; *significant value ( *p* < .05).

## Discussion

The overall prevalence of *H. pylori* infection found in the studied population was 28.7%. This in accordance to what might be expected, although the prevalence of *H. pylori* infection has decreased in some countries [24], it varies from country to country, affecting mainly the less affluent population [25]. Thus, Elitsur et al. have shown that *H. pylori* infection affected 12% of asymptomatic children in USA [12], whereas, in developing countries, this infection has shown seroprevalence rates higher than 40% in asymptomatic children [11].

The variables studied in the present work that had the significance of their associations with *H. pylori* infection retained in the multivariate model and therefore, could be considered as potential risk factors for that infection were: age, number of siblings, nursery attendance, living in paved road, and flush toilet.

Gender has not been identified as a relevant characteristic for *H. pylori* acquisition in this or in other studies which have investigated this infection in childhood [8,26], although, in adult populations, male gender has been significantly associated with *H. pylori* infection [27]. Similar to other findings [28], the current study showed *H. pylori* infection increasing with the children’ age. The fact that 62.3% of the *H. pylori* seropositive children identified in this study were younger than 8 years old supports the hypothesis that in developing countries the acquisition of *H. pylori* infection can occur in early childhood [25]. Once acquired, an untreated *H. pylori* infection can become lifelong [29] and lead to the development of atrophic gastritis and metaplasia, which are risk factors for the development of gastric cancer [30].

Although the education level of the mother is a socioeconomic indicator and is considered a reliable marker of the estimated level of household hygiene, this variable was not associated with the prevalence of *H. pylori* infection in our study. This indicator and other socioeconomic markers such as family income and parents’ occupation have been negatively associated with *H. pylori* infection in other studies [28]. However, another household hygiene marker that was studied in the current work was the absence of a flush toilet in the house. This absence was associated with infection, supporting the hypothesis that the fecal–oral route may be an important mechanism of transmission of this bacterium [31,32].

Although no significant association was found between treated piped water and *H. pylori* infection, the ingestion of contaminated water is considered a likely mode of acquisition, independent of socioeconomic status [33]. Many studies have investigated the influence of water source as an important risk factor for *H. pylori* acquisition [28,34]. In an adult Indian population, for example, the prevalence was significantly higher (88.2%) among subjects with low clean water index (CWI), when compared to those with high CWI (33.3%) [34].

Variables pertaining to food intake were also assessed. Although meat intake was significantly associated in the uncontrolled analysis, in the multivariable model, neither meat nor vegetable intake showed to be potential risk factors for acquisition of this infection. To the best of our acknowledge, there are no reports on the association of these variables with acquisition of *H. pylori* infection, although once these foods are contaminated because of inadequate hygiene, the bacteria may survive long enough to cause infection [35]. As reported by Hopkins et al., people who have uncooked vegetable consumption habits were more infected by *H. pylori* than those who do not have them [36].

Number of siblings and nursery attendance were positively associated with *H. pylori* infection in our study. These variables may reflect crowding, which has been reported as one of the major risk factors for *H. pylori* infection [37,38].

Unlike other studies, it was also investigated whether the house location (served by paved road or not) is a relevant factor for the acquisition of *H. pylori* infection. This variable was identified as a potential risk factor for infection, indicating that contaminated soil may also be a source of infection [39].

Concluding, this study supports the hypothesis that *H. pylori* infection in children is highly related to poor hygiene and crowded conditions. This may explain why this infection is more prevalent in nonaffluent countries and populations. Thus, improvement in hygiene habits and basic sanitary conditions could help this and other similar populations to decrease *H. pylori* infection prevalence in childhood.
